# Internal, external and repeated-sprint demands in small-sided games: A comparison between bouts and age groups in elite youth soccer players

**DOI:** 10.1371/journal.pone.0249906

**Published:** 2021-04-28

**Authors:** Richard Hauer, Paul Störchle, Bettina Karsten, Harald Tschan, Arnold Baca

**Affiliations:** 1 Centre for Sports Science and University Sports, University of Vienna, Vienna, Austria; 2 Department of Sport Science, Football Academy St. Pölten NÖ, St. Pölten, Austria; 3 European University of Applied Science (EUFH), Rostock, Germany; California State University San Marcos, UNITED STATES

## Abstract

This study investigated the activity profile during small-sided games (SSG) in elite youth soccer players. Internal load (IL) including heart rate (HR) and external load (EL) such as distance covered in different speed-zones (SZ) were collected from forty-eight players of three different teams (U15, U16, U18). The investigation included a total of eighteen 5vs.5 SSGs, each consisting of four 2-minute bouts on a 40x32m pitch during spring season. Total group results (n = 48) showed a reduction in total-distance (p = 0.001; $\eta_{p}^{2}$ = 0.12), high-intensity-running (p = 0.009; $\eta_{p}^{2}$ = 0.09), and low-intensity-running distance (p = 0.028; $\eta_{p}^{2}$ = 0.07) between bouts. Similarly, a reduction in the number of both acceleration-low (p = 0.001; $\eta_{p}^{2}$ = 0.12) and deceleration-high (p = 0.003; $\eta_{p}^{2}$ = 0.11) values was observed. Additionally, time spent in HR-zones 3 and 4 (p≤0.007; $\eta_{p}^{2}$ ≥ 0.10), increased, with a reduction in HR-zone 1 (p = 0.000, $\eta_{p}^{2}$ = 0.25). Age group comparison showed less distance covered in SZ 1 (p≤0.000; $\eta_{p}^{2}$ = 0.56) and greater deceleration-high values (p≤0.038; $\eta_{p}^{2}$ = 0.32) in U15 players compared to other age groups. Further, U15 showed lower values in low-intensity-running compared to U18 (p = 0.038; $\eta_{p}^{2}$ = 0.22). No age-related differences were found for IL and repeated sprint ability (RSA) values. The higher EL in younger age groups should be taken into account when implementing soccer specific SSGs. In addition, HRmean values between 80–85% of HRmax and RSA numbers, which are similar to match-play data, indicate SSGs as an effective training tool to prepare youth soccer athletes for the demands of competition.

## Introduction

In high performance sports, maximum adaptive benefits are achieved when the training stimuli are similar to those of the competitive demands [1]. Ford et al. [2] provided some evidence for a relationship between the amount of time spent in activities specifically designed to improve performance (deliberate practice) and a player’s level of achievement. Game-based training enables the integrated training of physical, technical, and tactical aspects and therefore contributes to the specific area of talent development [3, 4]. To quantify the physical demands of training and match-play, time-motion analysis is commonly used. The training load is typically presented as external load (EL), defined as the work done by the athlete (e.g. distance or speed) and the internal load (IL) which is the associated physiological or perception response (e.g. heart rate, perception of effort) [5].

Small sided games (SSGs) represent modified soccer games played on reduced pitch areas, often using adapted rules and involving fewer players compared to traditional soccer games [6]. SSGs are increasingly used as skill-based conditioning games to prepare the athletes for the physical demands of competition [7]. It seems that both sport-specific and traditional approaches are equally effective modes to develop aerobic fitness and match performance [8]. As physiological stress drives adaptations, exercise intensity is considered as one of the key variables influencing the training response [9]. Several studies have investigated the effects of different variables, such as: number of players [10, 11]; pitch dimensions [12, 13]; coach encouragement [14]; game rules [11]; ‘floater’ players [15]; or the timing of SSGs within the training session [16], on exercise intensity. Besides the intensity itself, the length of bout duration can also affect IL and EL during SSGs. Köklü et al. [17] analyzed different game formats and bout durations and concluded that intervals with short and continuous bout durations lead to a higher IL, and short and middle bout durations to a higher EL. In contrast Fanchini et al. [9], stated that bout duration has only a minimal impact on exercise intensity and no significant effect on technical actions, such as pass, dribbling, shoot, tackle, etc.

From a physiological point of view, soccer is an aerobic-based anaerobic sport [18]. The intermittent nature of soccer encompasses brief bouts of high-intensity-running and longer periods of low-intensity exercise [19]. However, these brief bouts of high-intensity-running tend to occur at critical moments of play such as ‘close to goal situations’, or shots on goal [20]. The ability of a player to quickly recover and subsequently reproduce similar high-intensity-running efforts is, therefore, a crucial component commonly defined as repeated sprint ability (RSA) [21]. Importantly, pertinent recent research has reported that RSA performance in young soccer players is related to their maturity status [22]. In this context, Sanchez et al. [23] expressed the requirement of adjusting the training load according to the player’s age, particularly in RSA, as physical performance and muscle response can be complementary variables to manage accumulated fatigue in different age categories.

Age-related differences in youth soccer have previously been analyzed for variables including fitness and match running performance [24, 25], physical capacities and their correlation with soccer related physical performance [26], and tactical behavior [27]. Interestingly most of the studies investigating SSGs either only assessed one age category or used different methodologies, which hinders comparison of results [28].

To-date, one study analyzed age-related differences in one SSG task. Rábano-Muñoz et al. [15] compared the physical demands of three different age groups (senior, U19 and U17) and the tactical component of including ‘floater’ players in the task. Physical differences between the teams, particularly between U17 and U19, were found, demonstrating that drill demands are determined by age. In short, greater performances with higher EL values with increasing age were observed. Increasing EL were found in total and relative distance covered, as well as accelerations and decelerations [15].

Changes of direction, with repeated accelerations and decelerations have a high priority on performance in modern soccer. Throughout professional soccer match play, 300 (> ±0.5 m∙s^-2^; duration > 0.5s) accelerations and decelerations are performed per half [29], and about 18% of the total distance covered during a soccer match is completed while accelerating or decelerating [30]. These energetically demanding accelerations [31] and eccentrically damaging decelerations [32] can be used to give more detailed information about the soccer specific workload athletes are exposed to [33].

To date, no reference data to age-related RSA performance in SSGs exists. The development of reference data requires work with the highest standards available. Recent research [33–35] in this context described the limitations of common tracking variables in team sports, and their shortcomings with special reference to Global Positioning (GPS), and Local Positioning Measurement Systems (LPM). In order to produce accurate and reliable data, we used the LPM system (Inmotio, Austria) which provides accurate position, average speed, and peak speed measurements even in maximal intensity soccer specific exercises [33].

As it is common practice to perform similar training exercises in different age categories in soccer academies, there should be a greater focus on the long-term development to improve performance and decrease risk of injury and overtraining in young athletes. Therefore, the aim of the present study was twofold i) to investigate age-related differences and the development of reference data in elite youth players using identical soccer specific conditioning exercises in U15, U16 and U18 players and ii) to investigate possible alterations of internal and external loads within a training session (between bouts) to improve the understanding of SSGs as conditioning games, particularly in elite youth players.

Based on the literature, for aim i) we hypothesized that the demands will be age-related and further expected a higher number of intensive actions in form of RSA bouts with increasing age. For aim ii) we hypothesized higher amounts in IL for younger players but higher amounts of EL with increasing age. Further, we expected a decrease of EL during the course of SSGs, particularly in younger players.

## Materials and methods

To investigate age-related differences of EL and IL in SSGs, physical performance variables from 18 training sessions in three different age categories (U15, U16, and U18) were collected and analyzed (six sessions per team). Sessions consisted of a standardized warm-up (25 min) with three blocks: activation (movement-based techniques), achieving thermogenic effects (soccer specific), and active dynamic stretching (movement preparation). The main exercise consisted of four 2-minute bouts of self-regulated game play of 4 vs. 4 field players + goalkeepers (a total of 5 vs. 5 SSG format). The passive recovery period between the active bouts consisted of two minutes. The SSGs were performed as ‘double-box’ games on 40 x 32 m (1280 m^2^) grass pitches resulting in 128 m^2^ of space per player, including goalkeepers. A ball was constantly available by prompt replacement when off the pitch or immediately after a goal, with the aim to maximize effective playing time [12]. To increase result reliability the following rules were developed and applied for all games: a) the SSGs were performed immediately after the described warm-up; b) no additional tactical requirements such as man-to-man defense or contact limitations were used; c) no strong coach feedback was allowed, and d) the same eight players had to complete the four bouts in one training session. Individual data was collected using the validated [36, 37] LPM technology (Inmotio, Lenzing, Austria). Players wore a LPM vest containing a transponder on the back and antennas on both shoulders, ensuring optimal transmission to the base stations. The sampling rate was 40Hz (1kHz/25 transponders).

### Subjects

Forty-eight elite youth outfield soccer players (age: 15.85 ± 1.2 yrs, height: 173.7 ± 8.3 cm, mass: 63.1 ± 9.8 kg) from three different teams: U15 (N = 15, age: 14.6 ± 0.5 yrs, height: 169.6 ± 9.9 cm, mass: 55.7 ± 8.9 kg), U16 (N = 16, age: 15.6 ± 0.5 yrs, height: 176.1 ± 7.1 cm, mass: 64.3 ± 9.1 kg), and U18 (N = 17, age: 17.2 ± 0.6 yrs, height: 175.1 ± 6.8 cm, mass: 68.5 ± 7.2 kg) participated in the present study. Players had approximately 8–13 years of soccer specific experience. Typically, they performed one match per week in the “ÖFB youth league”, the highest Austrian youth league, with six full days between matches (categorized using the “match day plus and minus” format [38]). These six days consisted of one day off (MD+1), one recovery session (MD+2), four training sessions (MD-4 to MD-1), and additional strength training on MD-4 (separate session) and MD-2 (combined session: gym workout followed by field session). Analyzed SSGs were performed on MD-4.

Permission for the study was provided by the ethics committee of the University of Vienna (Reference Number 00411) and conducted in accordance with the Declaration of Helsinki. All participants and their parents received an information letter and completed a written consent form.

### Procedures

To allow direct comparisons between-age groups, the time-motion variables were identical for all categories. Six speed zones (SZ) were used for analysis [39]: SZ 1 (0–6.9 km∙h^-1^), SZ 2 (7–9.9 km∙h^-1^), SZ 3 (10–12.9 km∙h^-1^), SZ 4 (13–15.9 km∙h^-1^), SZ 5 (16–17.9 km∙h^-1^) and SZ 6 (≥18 km∙h^-1^). Low-intensity-running (<13 km∙h^-1^), high-intensity-running (≥13 km∙h^-1^) and sprint-running (≥18 km∙h^-1^) were defined according to the demands of youth soccer and pitch size. Players had to overcome the respective speed threshold for a minimum of one second to start the recording of both, high-intensity-running and sprint-distance.

RSA was characterized in two ways. First, *repeated-sprint bouts* (B3) were defined as a minimum of three sprint efforts separated by less than 21 s recovery [21, 40]. Secondly, *successive sprints* (SSP) were defined as two or more sprints that occurred with less than 21 s recovery between efforts (adapted to Gabbett et al. [20]).

Accelerations and decelerations had to reach ≥ ±2 m∙s^-2^ respectively with a minimum required duration of 0.5 s [41] and they were categorized as low (< ±3 m∙s^-2^) or high (≥ ±3 m∙s^-2^). Heart rate (HR) was recorded (1 s intervals) using HR sensors located on the player’s chest (Polar Electro, Kempele, Finland). Scores were expressed as mean percentage of maximum HR, and time (s) spent in the individual heartrate zones (HRz) 1 to 4: HRz1 (<75% of HRmax), HRz2 (75–84% of HRmax), HRz3 (85–89% of HRmax), and HRz4 (≥90% of HRmax). At the beginning of the intervention, maximum HR was determined from a maximally exhaustive 20 m field-based shuttle test which demonstrates moderate to high (0.79–0.93) intraclass correlation coefficients [42]. All sessions were held at the same time of the day to avoid circadian rhythm. Participants were instructed to usual nutritional and exercise behaviours.

### Statistical analysis

All statistical analyses were performed with the IBM software SPSS Version 25 (SPSS, Chicago, IL, USA). Assumptions of normality were verified by Kolmogorov-Smirnow and Shapiro-Wilk test, as well as by histograms. A one-way repeated measures ANOVA was used to analyse age group differences for all recorded variables. Effect sizes were calculated as partial eta-squared ($\eta_{p}^{2}$) and categorized as small ≥ 0.01 < 0.10, moderate ≤ 0.10 < 0.25, large ≥ 0.25. Depending on whether there was homogeneity of variance or not Bonferroni and Games-Howell corrected post-hoc pairwise comparisons were used to identify differences between U15, U16, and U18 for total training session values. To understand differences over the course of training, a repeated measures ANOVA was performed and pairwise comparisons were used to identify the differences between the individual bouts. In the same manner, a repeated measures ANOVA and multiple comparisons were used to evaluate individual bout differences between U15, U16, and U18. Data is reported as mean ± SD unless stated otherwise. The significance level was set at p ≤ 0.05. All calculations are based on a 95% confidence interval (CI).

## Results

### Age group differences

Tables 1 and 2 demonstrate results for EL-parameters and IL-parameters respectively. Results are presented with regard to age groups and total training sessions. Statistically significant differences were identified between age groups for distance covered in SZ 1, low-intensity-running, and number of deceleration-high (≥ 3 m∙s^-^^2^). When compared to U16 and U18, U15 demonstrated less distance covered in SZ 1 (26.6 ± 5.8 m∙min^-1^) which was associated with a large ES (p ≤ 0.001; $\eta_{p}^{2}$ = 0.56). Compared to other age groups, the number of deceleration-high was greater in U15 players (U15: 2.9 ± 0.9 n∙min^-1^), which was associated with a large ES (p ≤ 0.047; $\eta_{p}^{2}$ = 0.31). The low-intensity-running distance covered was highest in U18 players (92.3 ± 3.8 m∙min^-1)^ with significant differences compared to U15 players. This was associated with a moderate ES (p = 0.038; $\eta_{p}^{2}$ = 0.22). No significant differences were found for other EL and IL parameters between age groups.

**Table 1:** External load parameters for the total training sessions, p-values and effect size.

Parameter	Total	U15	U16	U18	P	ES$\left(\eta_{p}^{2}\right)$
Sprints [n/min]	2.58 ± 0.65	2.70 ± 0.81	2.51 ± 0.62	2.55 ± 0.58	0.712	0.014
Total Distance [m/min]	139.64 ± 12.52	143.36 ± 14.11	135.63 ± 10.90	140.91 ± 12.42	0.206	0.065
High-intensity-running [m/min]	44.60 ± 9.10	42.60 ± 8.38	45.91 ± 8.68	44.74 ± 10.14	0.614	0.021
Low-intensity-running [m/min]	89.07 ± 7.68	83.32 ± 11.30^16, 18^	89.78 ± 5.13	92.34 ± 3.84	0.003*	0.222
SZ 1 [m/min]	34.75 ± 6.65	26.61 ± 5.79^16, 18^	36.46 ± 3.85	38.70 ± 4.08	0.000*	0.559
SZ 2 [m/min]	27.59 ± 3.21	27.91 ± 4.24	27.45 ± 3.02	27.50 ± 2.71	0.919	0.004
SZ 3 [m/min]	26.74 ± 4.42	28.81 ± 5.57	25.87 ± 3.82	26.14 ± 3.83	0.143	0.079
SZ 4 [m/min]	20.50 ± 4.24	21.45 ± 5.11	20.17 ± 3.95	20.15 ± 3.98	0.647	0.018
SZ 5 [m/min]	11.21 ± 4.73	10.71 ± 3.06	12.11 ± 5.90	10.71 ± 4.53	0.611	0.021
SZ 6 [m/min]	14.57 ± 4.63	12.00 ± 3.77	15.56 ± 3.52	15.40 ± 5.55	0.063	0.111
Acceleration low [n/min]	0.92 ± 0.34	0.87 ± 0.34	0.95 ± 0.39	0.92 ± 0.32	0.821	0.009
Acceleration high [n/min]	1.99 ± 0.59	2.27 ± 0.60	1.83 ± 0.48	1.93 ± 0.64	0.124	0.090
Deceleration low [n/min]	0.87 ± 0.34	1.00 ± 0.37	0.79 ± 0.38	0.85 ± 0.25	0.225	0.063
Deceleration high [n/min]	2.20 ± 0.81	2.90 ± 0.94^16, 18^	1.76 ± 0.50	2.12 ± 0.64	0.000*	0.312

Sprints (≥18 km∙h^-1^), low-intensity-running (<13 km∙h^-1^), high-intensity-running (≥13 km∙h^-1^), speed zones: SZ 1: 0–6.9 km∙h^-1^, SZ 2: 7–9.9 km∙h^-1^, SZ 3: 10–12.9 km∙h^-1^, SZ 4: 13–15.9 km∙h^-1^, SZ 5: 16–17.9 km∙h^-1^ and SZ 6: ≥18 km∙h^-1^, accelerations and decelerations low (< ±3 m∙s^-^^2^), and high (≥ ±3 m∙s^-^^2^) in number per minute (n/min). Parameters are presented as mean ±SD.

*Significant differences (P ≤ 0.05). Superscript numbers reflect the significant differences between respective age groups.

**Table 2:** Internal load parameter for the total training sessions, p-values and effect size.

Parameter	Total	U15	U16	U18	P	ES$\left(\eta_{p}^{2}\right)$
HRmean [%]	82.14 ± 6.08	82.67 ± 5.81	82.72 ± 4.51	81.13 ± 7.70	0.717	0.016
HRz1 [s/min]	13.84 ± 8.74	13.47 ± 9.44	13.48 ± 7.52	14.58 ± 9.91	0.927	0.004
HRz2 [s/min]	14.32 ± 6.75	13.49 ± 4.67	14.69 ± 7.36	14.60 ± 7.85	0.878	0.006
HRz3 [s/min]	14.41 ± 7.24	15.50 ± 8.76	14.61 ± 5.47	13.33 ± 7.84	0.726	0.015
HRz4 [s/min]	16.78 ± 13.62	17.54 ± 12.96	17.21 ± 13.33	15.70 ± 15.20	0.927	0.004

HRmean in % compared to HRmax. HRz1: (<75% HRmax), HRz2: (75–84% HRmax), HRz3: (85–89% HRmax), HRz4: (≥90% HRmax). HR results are presented as mean ±SD.

### Bout differences

Over the course of training a reduction in total distances covered was observed (p = 0.001; $\eta_{p}^{2}$ = 0.12). Our post-hoc analysis revealed differences between bout #1 and bout #3 + #4 (p ≤ 0.048) and bout #2 and #3 (p = 0.003) for total distance covered. In this context, high-intensity-running (p = 0.009; $\eta_{p}^{2}$ = 0.09) and low-intensity-running (p = 0.028; $\eta_{p}^{2}$ = 0.07) distances also decreased over the course of training. The post-hoc analysis showed high-intensity-running differences between bout #1 and #3 (p = 0.036) only. Similarly, low-intensity-running differences between bout #2 and #4 were observed (p = 0.041).

Comparison for distance covered in different SZ showed an increase with a moderate ES in SZ 1 (p = 0.000; $\eta_{p}^{2}$ = 0.16) and a decrease with small to moderate ES in SZ 2, 3 and 4 (p ≤ 0.014; $\eta_{p}^{2}$ ≥ 0.08). Pairwise comparison between bouts for SZ showed differences between bout #1 and #3 and #4 (p = 0.002) in SZ 1 and in SZ 3 (p ≤ 0.029). Differences between bout #1 and #3 and #4 (p ≤ 0.005), and bout #2 and #3 and #4 (p ≤ 0.024) were found in SZ 2. Post-hoc analysis in SZ 4 did not show any differences between bouts.

A reduction in acceleration-low (p = 0.001; $\eta_{p}^{2}$ = 0.12) and deceleration-high (p = 0.003; $\eta_{p}^{2}$ = 0.11) was observed over the course of training (Fig 2). The post-hoc analysis showed differences between bout #1 and others for acceleration-low (p ≤ 0.023) and differences between bout #3 and #1 and #2 for deceleration-high (p ≤ 0.022).

IL-parameters revealed an increase in %HRmean with a large ES over the course of training (p = 0.000; $\eta_{p}^{2}$ = 0.33). The post-hoc analysis showed differences between bout #1 and others (p = 0.000) and bout #4 and #2 and #3 (p ≤ 0.010). Similarly, a reduction of time spent in HRz1 with large ES (p = 0.000, $\eta_{p}^{2}$ = 0.25) and an increase in HRz3 and 4 with small to moderate ES (p ≤ 0.007; $\eta_{p}^{2}$ ≥ 0.10) were observed. Pairwise comparison showed differences between bout#1 and others (p ≤ 0.002) in HRz1 and bout #1 and #2 and #3 in HRz3 (p ≤ 0.019). Additionally, differences were observed between bout #1 and #2 and #4 (p ≤ 0.004) and bout #3 and #4 (p = 0.018) in HRz4 (S2 Table).

### Bout differences between age groups

**Fig 1:**
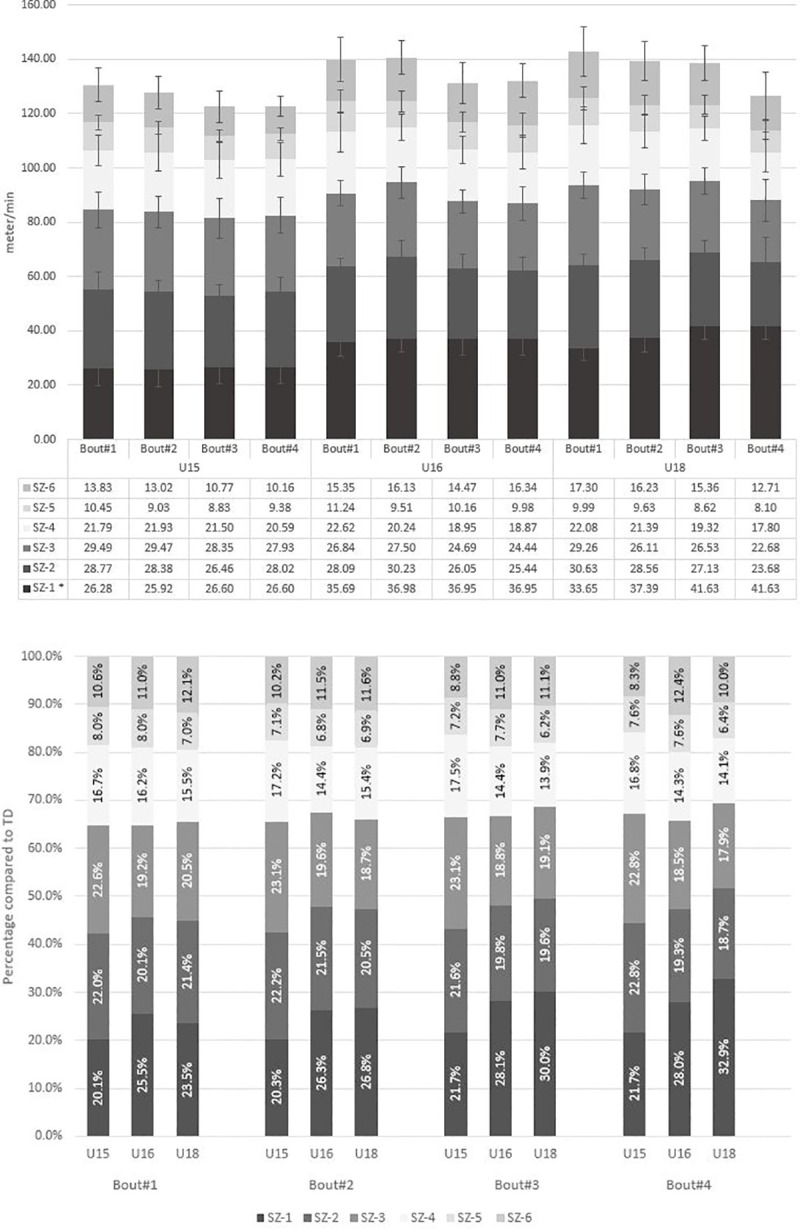
Distance covered in different speed-zones over the course of training concerning age groups. Panel A presents distance covered in different speed-zones as mean-values in m/min. Panel B shows % of distance covered in different speed-zones compared to total distance covered. SZ 1: 0–6.9 km∙h^-1^, SZ 2: 7–9.9 km∙h^-1^, SZ 3: 10–12.9 km∙h^-1^, SZ 4: 13–15.9 km∙h^-1^, SZ 5: 16–17.9 km∙h^-1^ and SZ 6: ≥18 km∙h^-1^. Dark grey colored bars stand for low-intensity-running (<13 km∙h^-1^, SZ 1–3) and light grey colored bars for high-intensity-running (≥13 km∙h^-1^, SZ 4–6) values. * Significant differences (P ≤ 0.05).

**Fig 2:**
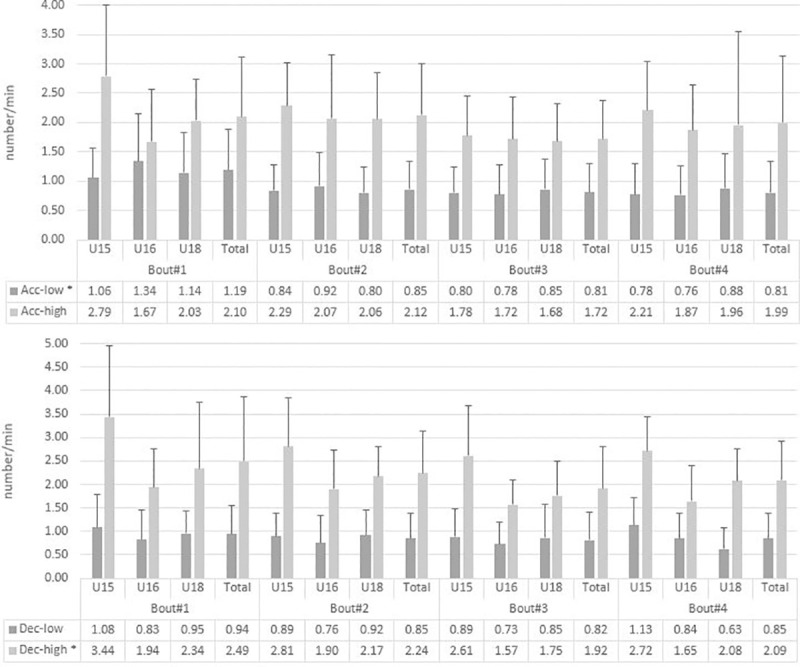
Number of low (< ±3 m∙s^-2^) and high (≥ 3 ± m∙s^-2^) acceleration and deceleration over the course of training concerning age groups. Panel A presents number of acceleration-low & high as mean-values in number/min. Panel B shows the number of deceleration-low & high as mean-values in number/min. * Significant differences (P ≤ 0.05).

Fig 1 provides an overview of distance covered in different SZ with Fig 1A showing results for distance covered in min^-1^ for bouts and age groups. Percentages of distance covered compared to the total distance covered in % are illustrated in Fig 1B. Results showed bout differences between age groups in SZ 1, low-intensity-running and deceleration-high. In each bout less distance was covered in SZ 1 in U15 players compared to other age groups (p ≤ 0.000; $\eta_{p}^{2}$ = 0.56). Further, different developments between bouts were found for age groups in SZ 1 (p = 0.000; $\eta_{p}^{2}$ = 0,19) (S1 Table). Similarly, U15 showed lower values in low-intensity-running distance compared to U18 (p = 0.038; $\eta_{p}^{2}$ = 0.22). Furthermore, U15 showed higher number of deceleration-high over the course of training (Fig 2B) compared to other age groups (p ≤ 0.038; $\eta_{p}^{2}$ = 0.32). Even though no significant differences between age groups and bouts were observed, it seems of interest that a 1:2 ratio between low-intensity-running:high-intensity-running distance remained constant over the course of training with no differences between age groups. We found contrasting HRmean values (p = 0.000; $\eta_{p}^{2}$ = 0.74, HRz1+2 and 3 p ≤ 0.031; $\eta_{p}^{2}$ ≥ 0.11) for U18 players compared to other age groups (S2 Table).

Fig 2 shows the amounts of accelerations and decelerations for each age group and complete collective per bout. Significant differences were found in acceleration-low and deceleration-high for the complete collective and deceleration-high between age groups over the course of training.

### Repeated sprints

The statistical analysis did not show differences between age groups (SSP: p = 0.210–0.687, $\eta_{p}^{2}$ = 0.02–0.07; B3: p = 0.068–0.930, $\eta_{p}^{2}$ = 0.00–0.12), bout differences (SSP: p = 0.323–0.599, $\eta_{p}^{2}$ = 0.02–0.04; B3: p = 0.203–0.686, $\eta_{p}^{2}$ = 0.02–0.14) for complete collective and bout differences between age groups (SSP: p = 0.691–0.917, $\eta_{p}^{2}$ = 0.01–0.03; B3: p = 0.391–0.886, $\eta_{p}^{2}$ = 0.01–0.21) for any repeated sprint variables. Table 3 provides an overview of SSP and B3 values for each age group and total group (n = 48) per training session. With no age group differences values for total group (n = 48) show an average of 3.1 ± 1.2 SSP bouts per trainings session. The bouts consisted of 2.7 ± 0.4 sprints per bout with a duration of 6.1 ± 1.3 s with 7.6 ± 2.6 s recovery between sprints. Results for B3 show 1.4 ± 0.9 bouts per training session consisting of 3.5 ± 0.5 sprints per bout. Sprint (6.1 ± 1.3 s) and recovery duration (8.1 ± 3.2 s) are similar to SSP values.

**Table 3:** Repeated sprint ability parameters for the total training sessions.

Age group	Parameter	Min	Max	Mean ± SD
**U15**	SSP_number_bouts_total	0.5	5.0	3.3 ± 1.0
	SSP_sprint/bout_total	2.0	3.6	2.7 ± 0.5
	SSP_duration_sprint_total [s]	4.0	7.3	6.0 ± 1.2
	SSP_duration_recovery_total [s]	4.4	12.9	7.3 ± 2.4
	B3_number_bouts_total	0.0	2.5	1.4 ± 0.8
	B3_sprint/bout_total	3.0	4.5	3.7 ± 0.5
	B3_duration_sprint_total [s]	4.3	7.7	6.3 ± 1.2
	B3_duration_recovery_total [s]	5.0	10.3	8.1 ± 1.6
**U16**	SSP_number_bouts_total	0.0	5.0	2.9 ± 1.2
	SSP_sprint/bout_total	2.0	3.3	2.7 ± 0.4
	SSP_duration_sprint_total [s]	3.9	8.9	6.5 ± 1.3
	SSP_duration_recovery_total [s]	5.1	13.4	8.1 ± 2.3
	B3_number_bouts_total	0.0	3.0	1.3 ± 0.8
	B3_sprint/bout_total	3.0	4.7	3.6 ± 0.6
	B3_duration_sprint_total [s]	4.1	9.0	6.2 ± 1.2
	B3_duration_recovery_total [s]	4.2	12.3	7.7 ± 2.6
**U18**	SSP_number_bouts_total	1.0	6.0	3.2 ± 1.4
	SSP_sprint/bout_total	2.0	3.5	2.6 ± 0.4
	SSP_duration_sprint_total [s]	2.6	9.3	5.8 ± 1.3
	SSP_duration_recovery_total [s]	2.6	14.5	7.5 ± 3.0
	B3_number_bouts_total	0.0	4.0	1.5 ± 1.0
	B3_sprint/bout_total	3.0	4.3	3.4 ± 0.5
	B3_duration_sprint_total [s]	3.9	9.1	5.9 ± 1.5
	B3_duration_recovery_total [s]	2.3	20.0	8.5 ± 4.3
**Total**	SSP_number_bouts_total	0.0	6.0	3.1 ± 1.2
	SSP_sprint/bout_total	2.0	3.6	2.7 ± 0.4
	SSP_duration_sprint_total [s]	2.6	9.3	6.1 ± 1.3
	SSP_duration_recovery_total [s]	2.6	14.5	7.6 ± 2.6
	B3_number_bouts_total	0.0	4.0	1.4 ± 0.9
	B3_sprint/bout_total	3.0	4.7	3.5 ± 0.5
	B3_duration_sprint_total [s]	3.9	9.1	6.1 ± 1.3
	B3_duration_recovery_total [s]	2.3	20.0	8.1 ± 3.2

(SSP = successive sprints; B3 = minimum of three sprints with < 21 s recovery). All data is presented as minimum, maximum and mean ±SD.

## Discussion

There is a distinct lack of knowledge about age-related differences for IL and EL-parameters for SSGs in the literature, even though research is rich with regard to the physical demands in soccer specific training and match-play [6, 19, 24]. Our results demonstrate less distance covered in SZ 1 for U15 players. In contrast, U15 showed a reduction in deceleration-high over the course of training but the total number of deceleration-high was significantly higher (U15: 2.9 ± 0.9 n∙min^-1^; p ≤ 0.047; $\eta_{p}^{2}$ = 0.31) compared to U16 and U18. These findings indicate that contrary to our hypothesis and the findings of Rábano-Muñoz et al. [15], who identified higher EL values with increasing age, EL was greater in U15 players. The suggestion of soccer being composed of stochastic interactions, i.e. the way and order actions occur during the game are chaotic [43], might offer a possible explanation for these contrasting results. This suggestion is further supported by Buchheit et al. [24] who speculated that this complexity of the game can affect a player’s actual movement activity. When compared to younger players and due to a greater level of experience, older players play a more tactically demanding game and therefore they tend to preserve some of their physical capacities (e.g.: total and high-intensity-running distance, accelerations and decelerations) [24].

With no differences in IL parameters, results suggest that our younger players were not physiologically overloaded. Explaining this, Ratel et al. [44] stipulated that younger players are able to resist fatigue when performing several repeated bouts of high-intensity exercise. It still seems questionable, however, whether the higher EL values might lead to a mechanical overload and thereby a higher risk of injury, even if the notion by Ratel et al. [44] holds true from a physiological point of view. In this context, Vanrenterghem et al. [45] proposed a new framework in which physiological and biomechanical load-adaptation pathways should be considered separately. For example, previous research has shown that reducing pitch size reduces the physiological load, but likely increases the biomechanical load [6, 46]. The biomechanical component of training load largely depends on propulsive and breaking forces against the ground [45]. In soccer, players undertake about 600 accelerations and decelerations in a single match [29]. These external kinetic demands of absorbing high forces from the impact with the environment and generating high forces to push off against the ground are responsible for the mechanical stresses on soft tissues [45]. This exercise induced muscular stress can be observed up to 120 hours post-match play [30]. If taking the higher amount of mechanical strain in form of deceleration-high in the younger players into account, different time frames between physiological and biomechanical adaptation should be considered, which may require adapted and adjusted periodization models between physiological and biomechanical loads with regard to different age groups [45].

To create soccer specific high-intensity endurance training, the five variables intensity, bout duration, recovery time between bouts, intensity during recovery bout, and total training duration (bout number x bout duration) have to be considered [17]. In this line Köklü et al. [17] analyzed different game formats (2:2, 3:3, 4:4) and bout durations (short: 6x2 min, middle: 4x3 min, long: 2x6 min, and continuous 1x12 min). Findings demonstrated that intervals with short, and continuous bout durations resulted in higher IL, while short and middle bout durations led to higher EL [17]. In the present study, EL and IL did change over the course of training regardless of age group. On the one hand, there is a reduction of total distance, high-intensity-running, low-intensity-running, and SZ 2+3 distance covered, which is in agreement with a lower number of acceleration-low and deceleration-high (S1 Table). As expected, IL increased over the course of training as %HRmean increased with a reduction of time spent in HRz1. Consequently, the time spent in HRz3 and HRz4 also increased (S2 Table). Contrary to our hypothesis, no age-related differences occurred. Most of the changes in the present study did not occur before the third SSG bout. This implies that 3 to 4 bouts of SSGs are required to induce physiological and mechanical responses. Our resultant 80–85% HRmax values demonstrate that the investigated SSG format is an adequate stimulus for aerobic training and that it can be used as intensive endurance training stimulus across age categories [15]. However, it is currently unclear if additional SSG bouts would bring additional positive or negative effects. One possible approach to determine an optimal number of bouts could be the use of thresholds derived from the most intensive periods of the game. As demonstrated in research, a smaller pitch size reduces most variables related to running speed and the total distance covered [12]. Therefore, running based parameters do not seem suitable for the use of thresholds in small and medium sized pitch training tasks. In their comparison between SSGs and the peak periods of official matches, Dalen et al. [47] showed that only acceleration based measurements can cover the requirements for the most intensive periods of the game. Results of the present study show a decrease over the course of training in acceleration and deceleration. These parameters should therefore be suitable for a threshold-based approach in medium sized pitch SSGs. However, more knowledge of the most intense periods in competitive soccer may be required to assist in the development of new specific training methodologies [48].

Research currently offers limited knowledge with respect to RSA in the context of SSGs. Mujika et al. [49] demonstrated a plateau in RSA when athletes reach U15. This is reflected in our results, as no differences in RSA values between age groups and bouts were identified. To gather further knowledge, this raises the need to provide information on the number and composition of RSA in SSG training formats. Results of our study, regardless of age group, show an average number of 3.1 ± 1.2 SSP bouts per training session. These bouts consisted of 2.0 to 3.6 sprints (mean 2.7 sprints) with a duration of between 2.6 and 9.3 s (mean 6.1 s) and recovery periods of between 2.6 and 14.5 s. (mean 7.6 s). The mean B3 total number of repeated-sprint bouts was 1.4 ± 0.9 with up to 4.7 sprints per bout. The respective minimum and maximum durations of the sprints were 3.9 and 9.1 s with recovery periods of between 2.3 and 20.0 s. These are very similar to SSP values. Position specific English Premiere League data for SSP of 1.7 to 5.2 bouts, 2.3 to 2.8 s duration, 6.7 to 8.1 s recovery (recovery ≤ 20 s separating efforts; velocities > 21 km∙h^-1^) [50], and RSA B3 characteristics in professional French soccer players of 1.1 ± 1.1 bouts per game, 3.3 ± 0.5 number of sprints per bout, duration 2.7 ± 0.7 s, and recovery periods of 13.6 s (recovery ≤ 20 s separating efforts; velocities > 19.8 km∙h^-1^) [51] have been reported. Our results show similar mean values for RSA efforts (SSP and B3), number of sprints per effort (only documented for B3) and recovery time between sprints (SSP). Although playing on a smaller pitch, the duration of the sprints shows distinctly higher values (mean 6.1 ±1.3 s) in the presented SSG format compared to the match data of mature players (< 3 s) [50, 51]. A reason for this difference could be the lower threshold velocity in our study (≥ 18 km∙h^-1^). However, our results indicate that medium sized SSGs are able to replicate the mean RSA demands of the game. In two out of eight players (22%) no B3 activities were found during training sessions (SSP activities are only affected in 3%), which requires consideration when using SSGs to prepare players for the RSA game demands. However, investigations into repeated sprint sequences in youth soccer matches showed that between 5 and 30% of players did not engage in any RSA activities [52].

Caution is required when comparing or interpreting training and match activities measured by different systems and settings [35]. Buchheit et al. [25, 52] suggested the use of individual thresholds to compare maturation states particularly with respect to high speed and repeated sprint demands. Our findings are limited as match data was not considered and a next step should therefore compare age-specific match data with training data. To allow easier direct comparisons between-age groups, the time-motion variables used in our study were identical for all categories. Although the LPM system is an accurate system to track distance, average speed, and peak speed of the players, Stevens et al. [33] showed that the accuracy for peak acceleration and deceleration is limited. However, when these error margins are kept in mind, the LPM system can be used to quantify average accelerations and parameters such as summed acceleration, as done in this study. Another limitation is that no tactical parameters were included in the measurements. From the perspective of the coach, conditioning exercises will always remain part of the playing style development process. Therefore, one criterion for conditioning games must be that the tactical guidelines can be maintained over the course of training.

In conclusion, no age-related differences in IL parameters were observed, but a higher amount of ELs in younger players was found. Therefore, the possibility of a mechanical overload and a higher risk of injury should be taken into account. Further, our results indicate that skill-based conditioning games offer an effective method to prepare young soccer players for the aerobic and repeated sprint demands of competition. New methodical approaches such as the one presented should further investigate the optimal number of bouts for conditioning SSGs.

## Practical applications

Our study provides novel insights into the IL, EL and RSA demands of SSGs in three different age categories in elite youth soccer players. Due to the similar IL but higher EL demands found, we suggest practitioners to consider physiological and biomechanical load-adaptation pathways separately. The resulting respective response rates have consequences for the planning of training and/or rehabilitation sessions to enhance performance and to prevent injuries, and therefore should be considered in the periodization model [45]. Our results additionally demonstrate that a 5-a-side SSG provides an adequate stimulus for aerobic training and therefore it can be used for soccer specific endurance training in elite U15, U16 and U18 players. Furthermore, a 2:1 relationship between low-intensity-running and high-intensity-running distances can be assumed when planning medium sized SSGs. Finally, to induce a required physiological and mechanical response, our results show that a minimum of 3 to 4 bouts may be required. In summary, our RSA findings indicate that players are able to replicate the mean RSA demands of the game, but coaches should be aware that in about 2 out of 8 players no RSA activities (as defined by Spencer et al. [40]) occurred during the course of training.

## Supporting information

S1 Table External load parameters per bout over the course of training concerning age groups and total group (n = 48). Sprints (≥18 km∙h^-1^), low intensity running (<13 km∙h^-1^), high intensity running (≥13 km∙h^-1^), speed zones: SZ 1: 0–6.9 km∙h^-1^, SZ 2: 7–9.9 km∙h^-1^, SZ 3: 10–12.9 km∙h^-1^, SZ 4: 13–15.9 km∙h^-1^, SZ 5: 16–17.9 km∙h^-1^ and SZ 6: ≥18 km∙h^-1^, accelerations and decelerations low (< ±3 m∙s^-2^), and high (≥ ±3 m∙s^-2^). All data is presented as mean ±SD.
(DOCX)

S2 Table Internal load parameters per bout over the course of training concerning age groups and total group (n = 48). HRmean in % compared to HRmax. HRz1: (<75%), HRz2: (75–84%), HRz3: (85–89%), HRz4: (≥90%). All data is presented as mean ±SD.
(DOCX)

S3 Table Repeated sprint ability parameters per bout over the course of training concerning age groups and total group (n = 48). All data is presented as mean ±SD.
(DOCX)
