# Supplementary material for: Internal, external and repeated-sprint demands in small-sided games: A comparison between bouts and age groups in elite youth soccer players
Source: PLoS One. 2021 Apr 28;16(4):e0249906. doi: 10.1371/journal.pone.0249906 (PMC8081179; doi:10.1371/journal.pone.0249906)
Supplement: S1 Table — Sprints (≥18 km∙h-1), low intensity running (<13 km∙h-1), high intensity running (≥13 km∙h-1), speed zones: SZ 1: 0–6.9 km∙h-1, SZ 2: 7–9.9 km∙h-1, SZ 3: 10–12.9 km∙h-1, SZ 4: 13–15.9 km∙h-1, SZ 5: 16–17.9 km∙h-1 and SZ 6: ≥18 km∙h-1, accelerations and decelerations low (< ±3 m∙s-2), and high (≥ ±3 m∙s-2). All data is presented as mean ±SD. (DOCX) [file pone.0249906.s001.docx]

**S1 Table.** External load parameters per bout over the course of training concerning age groups and total group (n= 48).

| **Parameter** | **Bout** | **Total** | **P** | **ES**$\mathbf{(}\boldsymbol{\eta}_{\boldsymbol{p}}^{\mathbf{2}}\mathbf{)}$ | **U15** | **U16** | **U18** | **P** | **ES**$\mathbf{(}\boldsymbol{\eta}_{\boldsymbol{p}}^{\mathbf{2}}\mathbf{)}$ |
| --- | --- | --- | --- | --- | --- | --- | --- | --- | --- |
| Sprints [n/min] | 1 | 2.73 ± 1.01 | 0.064 | 0.049 | 2.89 ± 1.18 | 2.65 ± 1.07 | 2.68 ± 0.85 | 0.875 | 0.017 |
|  | 2 | 2.69 ± 0.83 |  |  | 2.69 ± 0.92 | 2.63 ± 0.91 | 2.75 ± 0.72 |  |  |
|  | 3 | 2.46 ± 0.77 |  |  | 2.70 ± 0.91 | 2.28 ± 0.65 | 2.46 ± 0.77 |  |  |
|  | 4 | 2.48 ± 0.77 |  |  | 2.62 ± 0.88 | 2.48 ± 0.72 | 2.36 ± 0.78 |  |  |
| Total Distance [m/min] | 1 | 142.57 ± 16.58^3, 4^ | 0.001* | 0.117 | 148.75 ± 17.76 | 139.60 ± 17.35 | 141.07 ± 14.54 | 0.139 | 0.067 |
|  | 2 | 141.26 ± 13.25^3^ |  |  | 145.29 ± 13.24 | 140.59 ± 15.33 | 139.02 ± 10.88 |  |  |
|  | 3 | 135.77 ± 13.25^2^ |  |  | 139.62 ± 15.72 | 131.03 ± 10.84 | 137.72 ± 12.83 |  |  |
|  | 4 | 137.04 ± 14.75^1^ |  |  | 139.78 ± 14.39 | 131.28 ± 11.67 | 140.81 ± 16.62 |  |  |
| High intensity running [m/min] | 1 | 48.45 ± 15.52^3^ | 0.009* | 0.089 | 46.07 ± 12.48 | 49.22 ± 16.48 | 49.36 ± 17.02 | 0.755 | 0.020 |
|  | 2 | 45.91 ± 10.77 |  |  | 43.98 ± 11.67 | 45.88 ± 9.83 | 47.25 ± 11.35 |  |  |
|  | 3 | 42.63 ± 10.63^1^ |  |  | 40.66 ± 8.65 | 43.35 ± 11.87 | 43.29 ± 10.99 |  |  |
|  | 4 | 41.43 ± 13.46 |  |  | 39.70 ± 8.23 | 45.19 ± 11.37 | 39.05 ± 17.43 |  |  |
| Low intensity running [m/min] | 1 | 90.15 ±9.58 | 0.019* | 0.065 | 84.54 ± 14.95 | 90.62 ± 6.32 | 93.53 ± 5.28 | 0.061 | 0.087 |
|  | 2 | 90.86 ± 9.67^4^ |  |  | 83.77 ± 10.65 | 94.71 ± 10.06 | 92.06 ± 5.55 |  |  |
|  | 3 | 88.95 ± 10.22 |  |  | 81.41 ± 10.96 | 87.69 ± 7.18 | 95.29 ± 8.41 |  |  |
|  | 4 | 86.34 ± 10.85^2^ |  |  | 83.56 ± 11.21 | 86.09 ±7.17 | 88.49 ± 13.33 |  |  |

| SZ 1 [m/min] | 1 | 32.47 ± 6.41^3, 4^ | 0.000* | 0.156 | 26.28 ± 6.40 | 35.69 ± 4.93 | 33.65 ± 4.71 | 0.000* | 0.191 |
| --- | --- | --- | --- | --- | --- | --- | --- | --- | --- |
|  | 2 | 34.26 ± 7.22 |  |  | 25.92 ± 6.50 | 36.98 ± 4.64 | 37.39 ± 5.05 |  |  |
|  | 3 | 36.04 ± 8.13^1^ |  |  | 26.60 ± 5.90 | 36.95 ± 6.02 | 41.63 ± 4.92 |  |  |
|  | 4 | 36.23 ± 8.30^1^ |  |  | 27.61 ± 6.59 | 36.21 ± 5.31 | 42.13 ± 6.43 |  |  |
| SZ 2 [m/min] | 1 | 29.23 ± 4.52^3, 4^ | 0.001* | 0.130 | 28.77 ± 6.58 | 28.09 ± 2.73 | 30.63 ± 4.03 | 0.134 | 0.070 |
|  | 2 | 29.11 ± 5.00^3, 4^ |  |  | 28.38 ± 4.35 | 30.23 ± 5.98 | 28.56 ± 4.45 |  |  |
|  | 3 | 26.56 ± 4.54^1, 2^ |  |  | 26.46 ± 3.81 | 26.05 ± 5.19 | 27.13 ± 4.52 |  |  |
|  | 4 | 25.44 ± 6.91^1, 2^ |  |  | 28.02 ± 5.01 | 25.44 ± 4.61 | 23.68 ± 9.23 |  |  |
| SZ 3 [m/min] | 1 | 28.45 ± 5.27^3, 4^ | 0.001* | 0.110 | 29.49 ± 6.47 | 26.84 ± 4.72 | 29.26 ± 4.75 | 0.240 | 0.054 |
|  | 2 | 27.48 ± 5.86 |  |  | 29.47 ± 5.92 | 27.50 ± 5.79 | 26.11 ± 5.81 |  |  |
|  | 3 | 26.34 ± 5.49^1^ |  |  | 28.35 ± 7.28 | 24.69 ± 4.28 | 26.53 ± 4.87 |  |  |
|  | 4 | 24.68 ± 7.13^1^ |  |  | 27.93 ± 6.49 | 24.44 ± 6.36 | 22.68 ± 7.76 |  |  |
| SZ 4 [m/min] | 1 | 22.20 ± 6.62 | 0.014* | 0.077 | 21.79 ± 5.59 | 22.62 ± 7.39 | 22.08 ± 6.83 | 0.761 | 0.021 |
|  | 2 | 21.12 ± 5.76 |  |  | 21.93 ± 6.80 | 20.24 ± 4.85 | 21.39 ± 5.99 |  |  |
|  | 3 | 19.75 ± 5.37 |  |  | 21.50 ± 6.84 | 18.95 ± 4.95 | 19.32 ± 4.57 |  |  |
|  | 4 | 18.91 ± 6.59 |  |  | 20.59 ± 6.31 | 18.87 ± 6.17 | 17.80 ± 7.24 |  |  |
| SZ 5 [m/min] | 1 | 10.56 ± 3.87 | 0.053 | 0.051 | 10.45 ± 2.70 | 9.99 ± 4.36 | 9.99 ± 4.36 | 0.793 | 0.022 |
|  | 2 | 9.43 ± 3.33 |  |  | 9.03 ± 2.48 | 9.51 ± 3.81 | 9.63 ± 3.50 |  |  |
|  | 3 | 9.23 ± 3.29 |  |  | 8.83 ± 2.37 | 10.16 ± 3.66 | 8.62 ± 3.41 |  |  |
|  | 4 | 9.11 ± 3.65 |  |  | 9.38 ± 2.29 | 9.98 ± 4.48 | 8.10 ± 3.45 |  |  |
| SZ 6 [m/min] | 1 | 15.70 ± 8.09 | 0.189 | 0.032 | 13.83 ± 6.34 | 15.35 ± 8.08 | 17.30 ± 9.19 | 0.609 | 0.031 |
|  | 2 | 15.36 ± 6.53 |  |  | 13.02 ± 5.95 | 16.13 ± 6.12 | 16.23 ± 7.19 |  |  |
|  | 3 | 13.85 ± 6.89 |  |  | 10.77 ± 5.68 | 14.47 ± 7.67 | 15.36 ± 6.50 |  |  |
|  | 4 | 13.35 ± 7.16 |  |  | 10.16 ± 3.84 | 16.34 ± 6.16 | 12.71 ± 8.77 |  |  |
| Acceleration low [n/min] | 1 | 1.19 ± 0.69^2, 3, 4^ | 0.001* | 0.122 | 1.06 ± 0.51 | 1.34 ± 0.81 | 1.14 ± 0.68 | 0.766 | 0.022 |
|  | 2 | 0.85 ± 0.49^1^ |  |  | 0.84 ± 0.45 | 0.92 ± 0.58 | 0.80 ± 0.44 |  |  |
|  | 3 | 0.81 ± 0.48^1^ |  |  | 0.80 ± 0.44 | 0.78 ± 0.49 | 0.85 ± 0.52 |  |  |
|  | 4 | 0.81 ± 0.53^1^ |  |  | 0.78 ± 0.52 | 0.76 ± 0.51 | 0.88 ± 0.59 |  |  |
| Acceleration high [n/min] | 1 | 2.10 ± 1.01 | 0.090 | 0.046 | 2.79 ± 1.21 | 1.67 ± 0.89 | 2.03 ± 0.71 | 0.341 | 0.047 |
|  | 2 | 2.12 ± 0.89 |  |  | 2.29 ± 0.73 | 2.07 ± 1.09 | 2.06 ± 0.79 |  |  |
|  | 3 | 1.72 ± 0.67 |  |  | 1.78 ± 0.67 | 1.72 ± 0.72 | 1.68 ± 0.64 |  |  |
|  | 4 | 1.99 ± 1.14 |  |  | 2.21 ± 0.83 | 1.87 ± 0.77 | 1.96 ± 1.59 |  |  |
| Deceleration low [n/min] | 1 | 0.94 ± 0.60 | 0.684 | 0.010 | 1.08 ± 0.71 | 0.83 ± 0.63 | 0.95 ± 0.47 | 0.509 | 0.038 |
|  | 2 | 0.85 ± 0.54 |  |  | 0.89 ± 0.51 | 0.76 ± 0.57 | 0.92 ± 0.54 |  |  |
|  | 3 | 0.82 ±0.59 |  |  | 0.89 ± 0.60 | 0.73 ± 0.47 | 0.85 ± 0.71 |  |  |
|  | 4 | 0.85 ± 0.55 |  |  | 1.13 ± 0.58 | 0.84 ± 0.54 | 0.63 ± 0.45 |  |  |
| Deceleration high [n/min] | 1 | 2.49 ± 1.37^3^ | 0.003* | 0.109 | 3.44 ± 1.52 | 1.94 ± 0.81 | 2.34 ± 1.41 | 0.664 | 0.028 |
|  | 2 | 2.24 ± 0.89^3^ |  |  | 2.81 ± 1.03 | 1.90 ± 0.83 | 2.17 ± 0.63 |  |  |
|  | 3 | 1.92 ± 0.88^1, 2^ |  |  | 2.61 ± 1.07 | 1.57 ± 0.54 | 1.75 ± 0.74 |  |  |
|  | 4 | 2.09 ± 0.83 |  |  | 2.72 ± 0.73 | 1.65 ± 0.76 | 2.08 ± 0.69 |  |  |

Sprints (≥18 km∙h^-1^), low intensity running (<13 km∙h^-1^), high intensity running (≥13 km∙h^-1^), speed zones: SZ 1: 0-6.9 km∙h^-1^, SZ 2: 7-9.9 km∙h^-1^, SZ 3: 10-12.9 km∙h^-1^, SZ 4: 13-15.9 km∙h^-1^, SZ 5: 16-17.9 km∙h^-1^ and SZ 6: ≥18 km∙h^-1^, accelerations and decelerations low (< ±3 m∙s^-^²), and high (≥ ±3 m∙s^-^²). All data is presented as mean ±SD.

*Significant differences (P ≤ 0.05). Superscript numbers reflect the significant differences between respective bouts.
