# Supplementary material for: Internal, external and repeated-sprint demands in small-sided games: A comparison between bouts and age groups in elite youth soccer players
Source: PLoS One. 2021 Apr 28;16(4):e0249906. doi: 10.1371/journal.pone.0249906 (PMC8081179; doi:10.1371/journal.pone.0249906)
Supplement: S2 Table — HRmean in % compared to HRmax. HRz1: (<75%), HRz2: (75–84%), HRz3: (85–89%), HRz4: (≥90%). All data is presented as mean ±SD. (DOCX) [file pone.0249906.s002.docx]

**S2 Table.** Internal load parameters per bout over the course of training concerning age groups and total group (n=48).

| **Parameter** | **Bout** | **Total** | **P** | **ES**$\mathbf{(}\boldsymbol{\eta}_{\boldsymbol{p}}^{\mathbf{2}}\mathbf{)}$ | **U15** | **U16** | **U18** | **P** | **ES**$\mathbf{(}\boldsymbol{\eta}_{\boldsymbol{p}}^{\mathbf{2}}\mathbf{)}$ |
| --- | --- | --- | --- | --- | --- | --- | --- | --- | --- |
| HRmean [%] | 1 | 77.29 ± 9.82^2, 3, 4^ | 0.000* | 0.330 | 82.47 ± 6.39 | 81.75 ± 5.08 | 68.63 ± 10.01 | 0.000* | 0.744 |
|  | 2 | 82.25 ± 7.01^1, 4^ |  |  | 83.36 ± 7.67 | 83.27 ± 4.34 | 80.32 ± 8.49 |  |  |
|  | 3 | 83.67 ± 6.24^1, 4^ |  |  | 82.30 ± 5.29 | 82.68 ± 5.53 | 85.77 ± 7.35 |  |  |
|  | 4 | 85.35 ± 6.19^1, 2, 3^ |  |  | 82.56 ± 4.88 | 83.18 ± 4.30 | 89.78 ± 6.49 |  |  |
| HRz1 [s/min] | 1 | 23.98 ± 18.23^2, 3, 4^ | 0.000* | 0.250 | 13.39 ± 9.88 | 14.74 ± 8.99 | 42.41 ± 16.61 | 0.000* | 0.570 |
|  | 2 | 12.89 ± 13.40^1^ |  |  | 12.79 ± 11.63 | 11.92 ± 7.10 | 14.00 ± 19.35 |  |  |
|  | 3 | 11.30 ± 12.63^1^ |  |  | 14.32 ± 9.74 | 14.42 ± 12.56 | 5.53 ± 13.41 |  |  |
|  | 4 | 8.54 ± 8.36^1^ |  |  | 13.38 ± 8.33 | 12.88 ± 5.69 | 0.00 ± 0.00 |  |  |
| HRz2 [s/min] | 1 | 14.60 ± 9.18 | 0.424 | 0.020 | 13.49 ± 4.19 | 17.78 ± 10.87 | 12.12 ± 9.67 | 0.031* | 0.109 |
|  | 2 | 16.75 ± 12.79 |  |  | 11.53 ± 4.40 | 13.79 ± 8.64 | 24.13 ± 17.44 |  |  |
|  | 3 | 14.27 ± 14.53 |  |  | 13.61 ± 8.14 | 13.14 ± 8.81 | 16.01 ± 22.18 |  |  |
|  | 4 | 12.91 ± 11.04 |  |  | 15.36 ± 6.77 | 14.07 ± 6.97 | 9.70 ± 16.11 |  |  |
| HRz3 [s/min] | 1 | 9.75 ± 9.08^2, 3^ | 0.007* | 0.097 | 16.30 ± 9.31 | 12.61 ± 6.64 | 1.39 ± 3.15 | 0.002* | 0.166 |
|  | 2 | 14.71 ± 10.30^1^ |  |  | 16.14 ± 10.53 | 15.06 ± 8.45 | 13.18 ± 12.22 |  |  |
|  | 3 | 17.99 ± 14.81^1^ |  |  | 16.30 ± 9.18 | 14.38 ± 9.50 | 23.21 ± 21.17 |  |  |
|  | 4 | 15.22 ± 11.72 |  |  | 13.26 ± 7.74 | 16.44 ± 7.62 | 15.52 ± 17.23 |  |  |
| HRz4 [s/min] | 1 | 11.67 ± 14.34^2, 4^ | 0.000* | 0.179 | 16.82 ± 14.25 | 14.87 ± 14.57 | 4.09 ± 11.51 | 0.215 | 0.064 |
|  | 2 | 15.65 ± 16.02^1^ |  |  | 19.55 ± 14.89 | 19.23 ± 14.90 | 8.69 ± 16.63 |  |  |
|  | 3 | 16.43 ± 15.85^4^ |  |  | 15.77 ± 12.25 | 18.06 ± 13.78 | 15.24 ± 20.66 |  |  |
|  | 4 | 23.33 ± 18.93^1, 3^ |  |  | 18.01 ± 14.11 | 16.62 ± 14.27 | 34.78 ± 21.98 |  |  |

HRmean in % compared to HRmax. HRz-1: (<75%), HRz-2: (75-84%), HRz-3: (85-89%), HRz-4: (≥90%). All data is presented as mean ±SD. *Significant differences (P ≤ 0.05). Superscript numbers reflect the significant differences between respective bouts.
