# Supplementary material for: Internal, external and repeated-sprint demands in small-sided games: A comparison between bouts and age groups in elite youth soccer players
Source: PLoS One. 2021 Apr 28;16(4):e0249906. doi: 10.1371/journal.pone.0249906 (PMC8081179; doi:10.1371/journal.pone.0249906)
Supplement: S3 Table — All data is presented as mean ±SD. (DOCX) [file pone.0249906.s003.docx]

**S3 Table.** Repeated sprint ability parameters per bout over the course of training concerning age groups and total group (n=48).

| **Parameter** | **Bout** | **Total** | **P** | **ES**$\mathbf{(}\boldsymbol{\eta}_{\boldsymbol{p}}^{\mathbf{2}}\mathbf{)}$ | **U15** | **U16** | **U18** | **P** | **ES**$\mathbf{(}\boldsymbol{\eta}_{\boldsymbol{p}}^{\mathbf{2}}\mathbf{)}$ |
| --- | --- | --- | --- | --- | --- | --- | --- | --- | --- |
| B3_number_bouts | 1 | 0.24 ± 0.34 | 0.353 | 0.023 | 0.37 ± 0.54 | 0.19 ± 0.19 | 0.18 ± 0.23 | 0.215 | 0.064 |
|  | 2 | 0.21 ± 0.22 |  |  | 0.21 ± 0.20 | 0.18 ± 0.27 | 0.23 ± 0.19 |  |  |
|  | 3 | 0.18 ± 0.18 |  |  | 0.12 ± 0.16 | 0.19 ± 0.18 | 0.23 ± 0.19 |  |  |
|  | 4 | 0.16 ± 0.16 |  |  | 0.15 ± 0.16 | 0.18 ± 0.16 | 0.14 ± 0.16 |  |  |
| B3_sprint/bout | 1 | 1.76 ± 0.46 | 0.686 | 0.048 | 2.06 ± 0.27 | 1.95 ± 0.37 | 1.38 ± 0.43 | 0.276 | 0.252 |
|  | 2 | 1.80 ± 0.83 |  |  | 1.88 ± 0.53 | 1.60 ± 1.24 | 2.00 ± 0.00 |  |  |
|  | 3 | 1.89 ± 0.64 |  |  | 1.63 ± 0.18 | 2.05 ± 0.91 | 1.81 ± 0.38 |  |  |
|  | 4 | 2.02 ± 0.58 |  |  | 2.50 ± 0.00 | 1.75 ± 0.66 | 2.13 ± 0.48 |  |  |
| B3_duration_sprint [s] | 1 | 2.70 ± 0.73 | 0.203 | 0.140 | 3.08 ± 0.33 | 2.85 ± 0.60 | 2.34 ± 0.97 | 0.873 | 0.090 |
|  | 2 | 2.73 ± 1.10 |  |  | 2.94 ± 0.34 | 2.69 ± 1.63 | 2.66 ± 0.66 |  |  |
|  | 3 | 2.65 ± 0.61 |  |  | 3.19 ± 0.34 | 2.46 ± 0.61 | 2.63 ± 0.66 |  |  |
|  | 4 | 3.17 ± 0.74 |  |  | 3.40 ± 1.27 | 2.97 ± 0.65 | 3.31 ± 0.75 |  |  |
| B3_duration_recovery [s] | 1 | 3.09 ± 1.37 | 0.604 | 0.059 | 3.29 ± 0.43 | 3.19 ± 1.93 | 2.87 ± 1.04 | 0.557 | 0.172 |
|  | 2 | 3.86 ± 1.93 |  |  | 4.44 ± 0.16 | 4.34 ± 2.66 | 2.98 ± 1.18 |  |  |
|  | 3 | 3.85 ± 1.86 |  |  | 5.96 ± 3.09 | 3.32 ± 1.72 | 3.44 ± 0.89 |  |  |
|  | 4 | 3.60 ± 1.66 |  |  | 4.83 ± 0.53 | 2.87 ± 1.55 | 3.89 ± 1.98 |  |  |
| B3_number_sprints | 1 | 1.90 ± 0.67 | 0.110 | 0.044 | 2.09 ± 0.57 | 1.81 ± 0.76 | 1.85 ± 0.65 | 0.879 | 0.019 |
|  | 2 | 1.83 ± 0.72 |  |  | 1.86 ± 0.55 | 1.83 ± 1.00 | 1.81 ± 0.57 |  |  |
|  | 3 | 1.63 ± 0.62 |  |  | 1.67 ± 0.59 | 1.51 ± 0.61 | 1.69 ± 0.67 |  |  |
|  | 4 | 1.74 ± 0.60 |  |  | 1.67 ± 0.62 | 1.75 ± 0.47 | 1.79 ± 0.72 |  |  |

All data is presented as mean ±SD.

| SSP_number_bouts | 1 | 0.42 ± 0.30 | 0.515 | 0.017 | 0.50 ± 0.27 | 0.42 ± 0.38 | 0.35 ± 0.26 | 0.390 | 0.047 |
| --- | --- | --- | --- | --- | --- | --- | --- | --- | --- |
|  | 2 | 0.42 ± 0.25 |  |  | 0.40 ± 0.29 | 0.36 ± 0.25 | 0.48 ± 0.23 |  |  |
|  | 3 | 0.36 ± 0.26 |  |  | 0.34 ± 0.23 | 0.39 ± 0.29 | 0.34 ± 0.27 |  |  |
|  | 4 | 0.43 ± 0.28 |  |  | 0.43 ± 0.24 | 0.36 ± 0.29 | 0.49 ± 0.29 |  |  |
| SSP_sprint/bout | 1 | 1.44 ± 0.42 | 0.599 | 0.023 | 1.65 ± 0.35 | 1.44 ± 0.53 | 1.31 ± 0.33 | 0.578 | 0.060 |
|  | 2 | 1.43 ± 0.44 |  |  | 1.50 ± 0.56 | 1.32 ± 0.58 | 1.47 ± 0.23 |  |  |
|  | 3 | 1.50 ± 0.51 |  |  | 1.35 ± 0.42 | 1.62 ± 0.76 | 1.49 ± 0.33 |  |  |
|  | 4 | 1.34 ± 0.33 |  |  | 1.38 ± 0.47 | 1.38 ± 0.32 | 1.28 ± 0.27 |  |  |
| SSP_duration_sprint [s] | 1 | 3.22 ± 1.24 | 0.326 | 0.041 | 3.08 ± 0.39 | 3.22 ± 0.74 | 3.30 ± 1.82 | 0.857 | 0.026 |
|  | 2 | 2.82 ± 0.77 |  |  | 3.14 ± 0.76 | 2.65 ± 0.90 | 2.76 ± 0.67 |  |  |
|  | 3 | 2.84 ± 0.52 |  |  | 2.84 ± 0.52 | 2.92 ± 0.65 | 2.78 ± 0.45 |  |  |
|  | 4 | 2.91 ± 0.92 |  |  | 3.23 ± 1.30 | 2.76 ± 0.82 | 2.84 ± 0.74 |  |  |
| SSP_duration_recovery [s] | 1 | 3.42 ± 1.93 | 0.551 | 0.025 | 3.04 ± 1.32 | 3.86 ± 1.92 | 3.31 ± 2.30 | 0.254 | 0.096 |
|  | 2 | 3.81 ± 2.11 |  |  | 4.38 ± 1.28 | 3.06 ± 2.00 | 4.03 ± 2.54 |  |  |
|  | 3 | 4.08 ± 1.68 |  |  | 3.75 ± 2.44 | 4.74 ± 1.48 | 3.77 ± 1.77 |  |  |
|  | 4 | 3.64 ± 1.53 |  |  | 3.36 ± 1.98 | 3.06 ± 0.84 | 4.24 ± 1.53 |  |  |
| SSP_number_sprints | 1 | 1.83 ± 0.74 | 0.345 | 0.024 | 1.98 ± 0.69 | 1.66 ± 0.88 | 1.85 ± 0.65 | 0.792 | 0.024 |
|  | 2 | 1.80 ± 0.64 |  |  | 1.82 ± 0.54 | 1.81 ± 0.83 | 1.77 ± 0.55 |  |  |
|  | 3 | 1.64 ± 0.58 |  |  | 1.55 ± 0.56 | 1.69 ± 0.52 | 1.66 ± 0.68 |  |  |
|  | 4 | 1.77 ± 0.63 |  |  | 1.72 ± 0.70 | 1.74 ± 0.49 | 1.82 ± 0.72 |  |  |
